# A Systematic Review of the Effect of Retail Food Environment Interventions on Diet and Health with a Focus on the Enabling Role of Public Policies

**DOI:** 10.1007/s13668-019-00295-z

**Published:** 2019-12-03

**Authors:** Catherine L. Mah, Gabriella Luongo, Rebecca Hasdell, Nathan G. A. Taylor, Brian K. Lo

**Affiliations:** 1 School of Health Administration, Dalhousie University, Sir Charles Tupper Medical Building, 5850 College Street, 2nd Floor, PO Box 15000, Halifax, NS B3H 4R2 Canada; 2 Dalla Lana School of Public Health, University of Toronto, 155 College Street, 5th Floor, Toronto, ON M5T 3M7 Canada; 3 Division of Nutritional Sciences, Cornell University, 417 Savage Hall, Ithaca, NY 14850 USA

**Keywords:** Community food environment, Consumer food environment, Retail food environment, Intervention, Review, Diet, Consumer purchasing, Noncommunicable diseases, Health behavior, Public policy, Policy context

## Abstract

**Purpose of Review:**

Update the state of evidence on the effectiveness of retail food environment interventions in influencing diet and explore the underlying role of public policy, through a systematic review of population-level interventions to promote health in the retail food environment, including community and consumer environments. Diet-related outcomes included purchasing, dietary intakes, diet quality, and health including weight. We coded studies for enabling public policy levers underpinning the intervention, using two widely used conceptual frameworks.

**Recent Findings:**

Of 86 articles (1974–2018), the majority (58 articles, 67%) showed at least one positive effect on diet. Thirteen articles (15%) discussed natural experiments, 27 articles (31%) used a design involving comparison groups including 23 articles (27%) specifically describing randomized controlled trials, and 46 (53%) were quasi-experimental (cross-sectional) evaluations. Across the “4Ps” of marketing (product, promotion, placement, and price), promotion comprised the greatest proportion of intervention strategies, especially in earlier literature (pre-2008). Few studies combined geographic access interventions with 4P strategies, and few used robust dietary intake assessments. Behavior change communication remains an intervention mainstay, but recent work has also incorporated environmental and social planning, and fiscal strategies. More recent interventions were multi-component.

**Summary:**

The retail food environment intervention literature continues to grow and has become more robust overall, with clearer evidence of the effect of interventions on diet-related outcomes, including consumer purchasing, dietary intakes, and health. There is still much scope for development in the field. Attention to enabling public policy could help to strengthen intervention implementation and evaluation in the retail food environment.

## Introduction

Dietary factors are the leading modifiable risk for global morbidity and mortality [1, 2] and a problem of serious policy concern [3]. Growing epidemiological research has investigated how the food choice environment in communities contributes to diet [4, 5]. Retail food stores are the main community food source for many populations, central to food distribution in both advanced and developing economies [6]. Spatial analysis of the retail food environment shows mixed associations between geographic access to stores, diet, and health [5], and an important explanation is the multidimensional character of retail exposures [7], including the complex ways in which humans move and behave in their food environments. Theoretical frameworks distinguish the *community* food environment (distribution of stores in an area, and how shoppers encounter them through daily mobility) and the *consumer* food environment (attributes experienced by shoppers in-store, influences usually categorized by the 4Ps of marketing: product, promotion, placement, and price) [8]. Variation among retailers in consumer environment features, such as product availability and price [9, 10], can modify associations between the community food environment and diet [4, 5].

### Background: a Brief Review of Retail Intervention Reviews

Within this context, growing attention has been paid to intervention strategies to reduce population dietary risk related to retail food environment exposures [11, 12]. Table 1 describes existing systematic reviews of interventions in community and consumer retail food environments to shape diet and health.

**Table 1:** Summary of recent systematic reviews on the effectiveness of retail food environment interventions in community and consumer environments, 2012-2018

1st author	Year	*N*	Settings and interventions included	Main outcome	Dates included
Reviews specifically focused on retail food environment interventions (direct evidence)					
Adam [13]	2016	42	Physical retail food store interventions related to obesity and to increase the consumption of healthy foods, including price, information, and access/availability	Sale/purchase of healthy foods	2003 to 2015
Cameron [14•]	2016	49	Supermarket-based interventions, including product, promotion, and placement	Food purchasing, dietary intake, and weight	Database inception to December 2015
Escaron [15]	2013	58	Supermarket and grocery store-based interventions, including point-of-purchase information, price, availability, promotion, and advertising	Consumer awareness, use, knowledge and beliefs, preferences, sales, and process measures	Late 1940s to July 2012
Gittelsohn [16]	2012	16*	Small store (< 10 employees and < 1,000 sq ft) interventions to influence food access and consumption	Process measures, store impact, consumer psychosocial and behavioral impact, consumer health	1990 to September 2010
Glanz [17]	2012	125	Food marketing confronted by consumers in grocery stores, including product, placement, price, and promotion; lab experiments, observational, and field interventions included	Food purchases and/or consumption	1995 to 2010
Hartmann-Boyce [18]	2018	55	Settings and interventions included: Supermarket and convenience store interventions including simulations, including price or rewards, placement, promotion, information, and swaps, randomized controlled trials only	Consumer purchasing	No date limit (search carried out June 2017)
Hasanthi-Abeykoon [19]	2017	11	Newly opened grocery stores, with or without added in-store intervention components	Physical or psychological health, psychological factors, food security, dietary intake, food purchasing, other food behavior	1995 to November 2015
Liberato [20]	2014	32	Nutrition interventions at the point-of-sale, including availability, affordability, or nutrition education/promotion	Food purchasing or dietary intake	No date limit (article published September 2014)
Pinard [21]	2016	19	Retail food environment research in small food stores, including observational studies as well as interventions, and focus on rural	No limits	May 2005 to May 2015
van’t Riet [22]	2012	16	Product health information presented at the point-of-purchase	Food sales or purchasing	1980 to 2010
Woodruff [23]	2017	23	Initiatives to increase spatial access to food retailers	Fruit and vegetable consumption among adults	Database inception to November 2015
Reviews on population health policies with influences in retail store settings (indirect evidence)					
Afshin [24]	2015	N/R	Broad range of policy interventions (mass media, labels, school procurement, worksite wellness, community built environment, fiscal, marketing) directed towards healthier dietary behavior and diet-related risk factors for cardiovascular disease	Dietary intake, adiposity, blood pressure, and blood lipids	1980 to N/R (article published September 2015)
Allender [25•]	2012	N/R	Quantitative primary evidence of the relationship between nine policy areas intended to improve environments for healthy eating and physical activity at the local government level; and nutrition, physical activity, or weight	Summary of evidence was not reported; was used as the basis for qualitative research with informants	N/R; some sub-searches were limited to within last 10 years
Thow [26]	2014	43	Fiscal policies to encourage healthy diets (sugar-sweetened beverage, fat, and calorie-based taxes; nutrient profiling taxes; and healthy food subsidies). Only 4/43 papers assessed an actual tax or subsidy vs. model/hypothetical	Consumption including purchasing and dietary intake	January 2009–March 2012

N = number of included papers

*N/R* not reported

*This review used grey literature as well as peer-reviewed academic literature, and reported *n* as number of trials

These reviews have demonstrated the substantial heterogeneity among interventions [20], but also show collectively that methodologies for evaluating interventions have strengthened over time, with corresponding clearer effects on food selection behavior, especially purchasing. The 2016 review of supermarket interventions by Cameron et al. [14•] in this journal found that 70% of interventions reported a positive (healthy) effect on food purchasing. The magnitude of effect differed widely, however, and some (generally weaker) studies demonstrated no effect.

Intervention strategies for smaller (i.e., convenience) versus larger (i.e., supermarkets) stores have tended to be assessed separately [14•], although important commonalities emerge when the literature is grouped. Escaron et al. [15] and Gittelsohn et al. [16] both concluded that the evidence for altering the retail choice architecture through multi-component interventions was stronger than for single component interventions, such as changing prices alone, or education/labeling alone.

Within systematic reviews to date, an understudied feature is the public policy context in which interventions are implemented [16]. Realizing the full implementation and impact of population health interventions in community settings requires enabling public policies led by government, a core principle of healthy public policy [27]. Three reviews have assessed public policy related to retail food environment interventions, summarized in Table 1. Allender et al. [25•] started with a review of health evidence but did not report it in the article, focusing instead on acceptability and feasibility of interventions as well as other policy and political considerations, through a local jurisdictional case. Allender et al. [25•] noted that their paper was addressing a key gap in population health literature that articulates intermediary, but necessary steps for policy change, where changes are appraised within a legal architecture and policy process. Afshin et al. [24] and Thow et al. [26] took a more macro approach, focusing specifically on systems-level policy interventions that might have an effect in the community built environment and retail stores, such as food subsidies/taxes.

No review of retail food environment interventions to date has assessed directly how the evidence of effectiveness of interventions is linked to their policy salience. Yet we would argue that this is essential to advance our understanding of how policy can enable successful interventions [11]. For instance, it is widely accepted that retail food environments in publicly funded institutions (e.g., schools, hospitals, recreation facilities) should be governed by supportive government policies that set the conditions for successful retail implementation and consumer uptake of healthier food options. Nutrition researchers are also increasingly examining how upstream regulatory approaches targeting food manufacturers can be used to accomplish public health goals. Only a highly limited range of healthy public policy proposals have been proposed to date for the domain of the private sector retail store, such as zoning. So as a starting point for greater research attention to the diverse policy instruments that might be used, in the current review, we were interested in expanding our understanding of the *policy assumptions* underlying the body of research on retail interventions designed to shift population diets. Like Allender, our aim is to connect interventions in a more direct way to government policy structures. This is especially important for the retail food environment, where a breadth of policy levers, government authorities, diverse private sector actors, and the governance and relational features among them make up the linking steps to a healthier population diet.

The objective of this paper was thus two-pronged: (a) update the state of the evidence on effectiveness of community and consumer food environment interventions in influencing diet (3 years has passed since the end date of literature captured in Cameron’s review [14•], which also focused solely on supermarkets, and did not include fiscal interventions) and (b) begin to explore the underlying role of public policy in these interventions.

## Methods

### Search Strategy

Working with an academic librarian at our institution, a systematic search of published peer-reviewed research literature was conducted in PubMed, Scopus, and CINAHL, published from the beginning of each database through to November 2018. The same forwards search was used for each database (* = truncation Boolean operator):

- Retail food outlet: food environment OR food retail OR grocer* OR food store OR convenience store OR food market OR supermarket* OR gas station
- Intervention foci: price OR pricing OR promotion OR intervention* OR program* OR initiative* OR evaluat* OR marketing
- Outcomes:nutrition OR diet OR health* OR chronic disease OR food choice OR food purchasing OR obes* OR overweight OR body weight

Reference lists from seven of the existing systematic reviews [14•, 15, 16, 19–21, 28] were then hand-searched to identify any articles that may have been missed (backwards search).

### Inclusion Criteria

We included original peer-reviewed articles in English, with full-text available. Articles were eligible if the intervention aimed to promote health in the retail environment at the population-level. Interventions had to be implemented within real-world retail outlets, defined as fixed location commercial establishments with the main purpose of the business being the sale of a product line(s) of food and non-alcoholic beverages, including grocery stores, supermarkets, convenience stores, and gas stations. A field experiment involving nutrition labels affixed to supermarket shelves was eligible [29]; experiments conducted in purpose-built mock store laboratories were not. The retail literature does not use the terms “grocery stores” and “supermarkets” interchangeably, so we accepted each term as presented by the author(s). Interventions could be evaluated with or without a comparison group and could use a quantitative, qualitative, or mixed methods approach. Interventions could include changing the availability or mix of retailers in a geographic area (community food environment) or the “4Ps” in-store, including product, pricing, placement, or promotion of food and non-alcoholic beverages (consumer food environment). Interventions could be interactive (e.g., dietitian consultations) or non-interactive (e.g., shelf labels).

Price interventions were included in the review as long as the general population of shoppers entering the store was eligible for having the intervention applied. For example, a study restricting participation to shoppers meeting a body mass index (BMI) criterion [30] was excluded. Other pricing interventions relied on cohort enrolment prior to allocation, involving a store loyalty card system to receive discounts [31, 32]; we considered these ineligible, as they targeted members rather than the general population. All dietary outcomes were eligible, including subjective or objective measures, and encompassing purchasing, dietary intake, diet quality, or diet-related health including weight. We did not place any restrictions by administrative jurisdiction or geography. Explicit reference to enabling public policy was not a factor for inclusion or exclusion, but explicit references were coded for in the policy analysis of included articles.

### Exclusion Criteria

Interventions were excluded if they were implemented in organizational or foodservices environments (e.g., restaurants, fast-food outlets, vending machines, schools, workplaces). Although the distinction between store and foodservices business models is increasingly blurred (e.g., grab-and-go café outlets selling own product lines; supermarkets that offer ready-to-eat items including dine-in), it is still reasonable to exclude foodservices from this study. Foodservices outlets’ main objective is *serving* of food for immediate consumption, versus *sale* of food per se. This is reflected in government licensing arrangements for foodservices outlets which are typically a different category from stores and may entail different enforcement requirements. Food product reformulation without a clear retail component was also excluded [33], as were price interventions external to the retail environment such as mass media-distributed store vouchers. We excluded mobile and online shopping, farmers markets, and primarily non-food retailers such as pharmacies. Formative evaluations, protocols, preliminary planning documents, opinion pieces, and systematic reviews were excluded. As noted above, experiments conducted “in vitro” in lab or web purchasing simulations [34] were also excluded.

### Study Selection

**Fig. 1:**
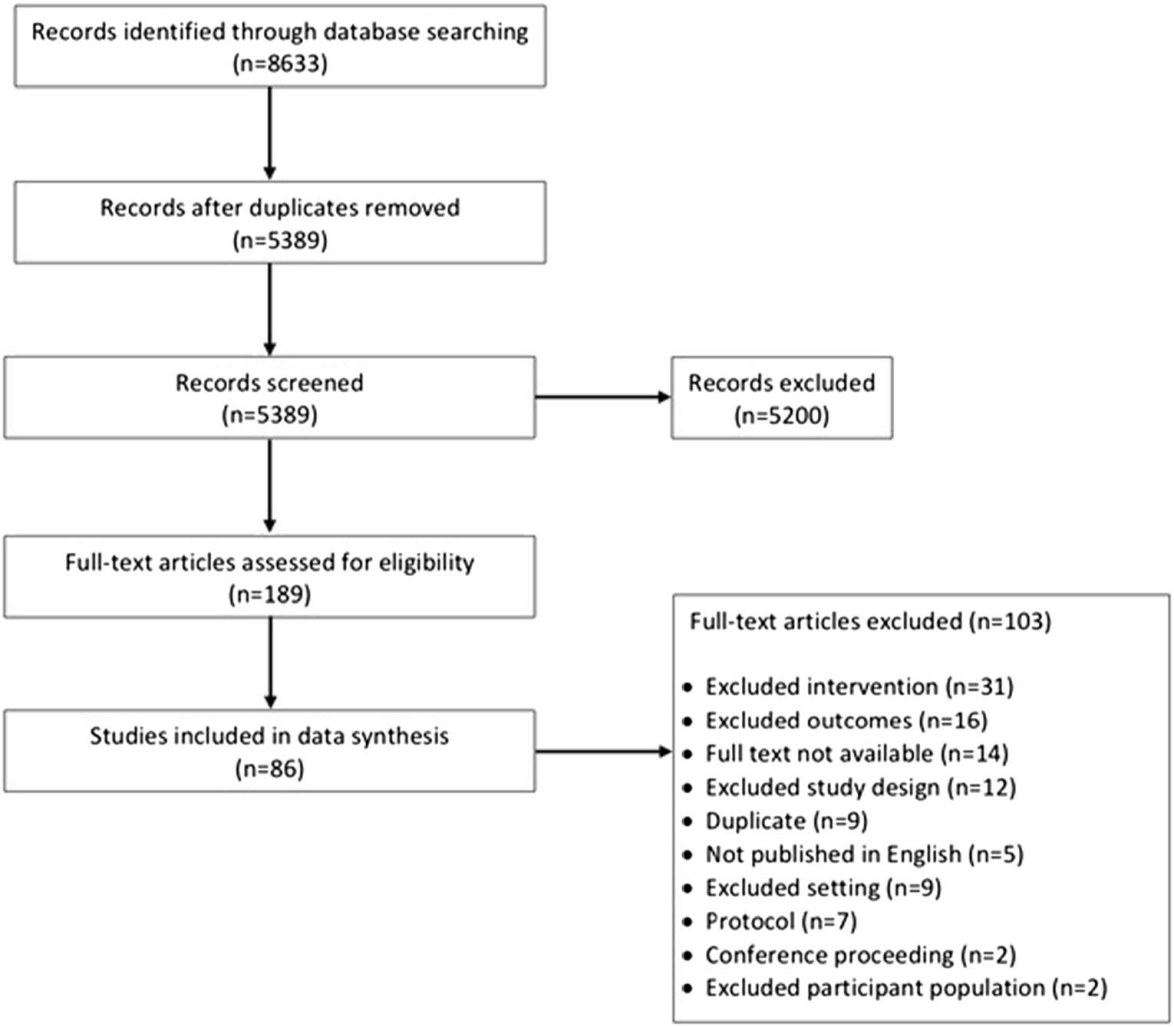
PRISMA flow diagram, review of retail food environment interventions in community and consumer environments, 1974–2018

Studies extracted from the databases were uploaded to the Covidence web application, which removed the vast majority of duplicates. Other duplicates were hand-searched and manually removed. Using Covidence, six reviewers independently screened 5,389 articles by title and abstract, with each decision to include or not requiring agreement between at least two reviewers. Studies then underwent full-text screen, with decisions again requiring two reviewers. Reviewers met by phone to discuss conflicts, and if necessary, a third reviewer was consulted to resolve the conflict. For the backwards search, each team member reviewed the reference list of one of the reviews and assessed papers for eligibility; titles of the references were initially screened, then full-text for eligible references were compiled and reviewed for inclusion. Where an article included from the backwards search had been previously excluded through the forwards search, two reviewers resolved the discrepancy (Fig. 1).

### Data Extraction

Three coders co-designed the extraction tool and independently extracted study data, one coder per article. To attend to rigour in data extraction and coding, the same three researchers who completed data extraction also completed policy coding. Although each article had a single coder, we integrated an iterative process of peer debriefing to arrive at findings. Coders deliberated throughout extraction and writing: (a) once to refine and finalize the extraction tool based on extracting at least one article each; (b) twice to further calibrate use of the tool; and (c) iteratively through the remainder of extraction, interpretation, and writing. Conflicts were resolved through consensus discussion.

In addition to basic extraction of study features, each article was coded deductively using two public policy frameworks. The Behavior Change Wheel [35, 36] was used to code for the behavioral mechanisms underpinning each intervention and associated types of enabling policies. The Behavior Change Wheel is a widely adopted conceptual framework for health intervention research that notably synthesizes 19 other theory-informed frameworks and goes beyond an exclusive focus on social cognitive or social ecological theory [35]. The Behavior Change Wheel has seven policy types (communications/marketing, guidelines, fiscal measures, regulation, legislation, environmental/social planning, and service provision) that map from “functions,” or how the intervention changes behavior. For example, fiscal policies (e.g., price subsidies on healthier foods) map five functions: incentivization, coercion, training, environmental restructuring, and enablement. The World Cancer Research Fund NOURISHING framework was then used to code for 10 types of public policies within three domains—food systems, food environments, and behavior change communication [12]. The NOURISHING framework is a widely used typology for governments to assemble a coherent suite of policies to support healthier diets [12]. Interventions could map to more than one policy type. We also coded for level of jurisdiction of policy levers, in terms of order of government likely responsible: local/municipal, provincial/regional/state/territorial, and national/federal, acknowledging interjurisdictional differences in authority. We followed a broadly Anglo-American federal political tradition where public policy levers such as urban planning would be local and packaged food labeling (related to the criminal law power) would be national. Corporate (organizational/store) policy was not analyzed in this review. An initial exploration of quality of the included articles was conducted using parameters of the Effective Public Health Practice Project quality assessment tool [37]; studies were ultimately not evaluated for quality, and not included or excluded based on quality, due to wide variation in methods limiting meaningful quality comparisons.

## Results

### Features of the Included Articles

We included *n* = 86 articles in the final review, covering 80 interventions. Most (69%) studies were from the USA. The remainder were from (descending order of proportion) Netherlands (6%) [38–42], UK (6%) [43, 44, 45••, 46–47], Australia (5%) [48–51], Canada (5%) [52–55], Denmark (3%) [56–58], Finland (2%) [59, 60], Sweden (1%), Belgium (1%) [61], Japan (1%) [62], and Norway (1%) [63].

The vast majority of articles (88%) reported on interventions in supermarkets (59 articles), grocery stores (14 articles), or a combination of these (3 articles). The remainder were in convenience stores (8 articles), convenience with supermarkets (1 article), and convenience with grocery (1 article). Thirteen articles (15%) discussed natural experiments. Twenty-seven studies (31%) used a design involving comparison groups (indicated as “RCT+” in Table 2): for synthesis purposes, we included in this category two articles using quasi-experimental designs with matched groups of stores allocated to intervention or comparison, one trial with comparison groups that did not mention randomization, and randomized controlled trials (24 articles, 27%). Forty-five (52%) articles were on quasi-experimental (cross-sectional) evaluations without comparison groups, including post-test only or repeat cross-sections. Only two of the convenience store studies used comparison groups, and they were among the more recent.

**Table 2:** Summary of strategies, effect on diet, and policy levers of retail food environment interventions in community and consumer environments, 1974–2018

1st author	Year	Effect	Geog	Price	Prod	Prom	Place	Study design	Intervention length	Outcome(s)	BCW policy levers	Jurisdiction
Curhan [64]	1974	Mixed^^		R		R	R	QE	7 months	$	Guidelines	National
Jeffery [65]	1982	Null				R		RCT+	6 months	$	Guidelines, communication/marketing	Multilevel
Levy [66]	1985	Positive				R		RCT+	2 years	$	Guidelines	State
Ernst [67]	1986	Null				R		QE	1 year	$	Guidelines, communication/marketing	National
Russo [68]	1986	Mixed^^				R		QE	33 weeks	$	Communication/marketing	National
Achabal [69]	1987	Null				R		QE	12 weeks	$	Guidelines	National
Dougherty [70]	1990	Null				R		QE	6 weeks	$	Guidelines, Communication/marketing	National
Scott [50]	1991	Positive				R	R	QE	15 weeks	D	Communication/marketing, environmental/social planning	Local
Winett [71] *Winett 1991b, Winett 1997	1991a	Positive				R		RCT+	10–36 min	$	Communication/marketing	National
Winett [72] *Winett 1991a, Winett 1997	1991b	Positive				R		RCT+	8–32 min	$	Communication/marketing	National
Patterson [73]	1992	Null				R		RCT+	2 years	$	Guidelines, communication/marketing	State
Crawford [52]	1993	Positive				R		QE	1.5 h	$	Communication/marketing	State
Rodgers [74]	1994	Mixed^				R		QE	2 years	$, D	Communication/marketing	National
Paine-Andrews [75]	1996	Positive		R		R		QE	9.5 h	$	Guidelines, communication/marketing	Local
Anderson [76]	1997	Positive		R		R		RCT+	10 weeks	$	Fiscal measures	Multilevel
Kristal [77]	1997	Null		R		R		RCT+	8 months	Q	Fiscal measures, communication/marketing	Local
Teisl [78]	1997	Positive				R		QE	3 years	$	Guidelines, communication/marketing	National
Winett [79] *Winett 1991a, Winett 1991b	1997	Positive		R		R		RCT+	30–50 min	$	Communication/marketing	National
Narhinen [60] *Narhinen 2000	1999	Null				R		QE	12 weeks	$	Guidelines, communication/marketing	Local
Narhinen [59] *Narhinen 1999	2000	Positive				R	R	QE	12 weeks	$	Guidelines, communication/marketing	Local
Connell [80]	2001	Positive				R		RCT+	4 weeks	D	Communication/marketing	National
Weinehall [81]	2001	Positive				R		N	10 years	$	Guidelines	National
Wrigley [46]	2003	Positive	R					N	1 year	D	Environmental/social planning	Local
Steenhuis [40]	2004	Null				R		RCT+	6 months	D	Guidelines, communication/marketing	Multilevel
Wang [82]	2007	Null	R					N	6 months	D	Environmental/social planning	Multilevel
Cummins [44] *Cummins 2008b	2008a	Null	R					N	1 year	$	Environmental/social planning	Local
Cummins [45•] *Cummins 2008a	2008b	Null	R					QE	1 year	D, H	Environmental/social planning	Local
Song [83]	2009	Positive			R	R		QE	10 months	$	Guidelines, fiscal measures, communication/marketing	Local
Freedman [84]	2010	Null				R		QE	5 weeks	$	Guidelines, communication/marketing	National
Berning [29]	2010	Negative				R		RCT+	4 weeks	$	Legislation	National
Gittelsohn [85]	2010a	Mixed^^			W	R		QE	9–11 months	D	Service provision	Local
Gittelsohn [86]	2010b	Positive		R	W	R		QE	10 weeks	D	Service provision, fiscal measures, communication/marketing	Local
Jetter [87]	2010	Positive			R,W			QE	7 months	$	Service provision	Local
Sutherland [88]	2010	Positive				R		N	2 year	$	Guidelines, communication/marketing	National
Ogawa [62]	2011	Positive				R		RCT+	60 days	$	Guidelines, communication/marketing	Multilevel
Sigurdsson [63]	2011	Positive				R	R		2–4 days	$	Legislation	National
Dannefer [89]	2012	Null			R	R	R	QE	5 months	$	Guidelines, service provision, regulation, communication/marketing, environmental/social planning	Local
Holmes [90]	2012	Mixed^				R		QE	12 weeks	$	Guidelines	Multilevel
Milliron [91]	2012	Null				R		RCT+	4 months	$, Q, H	Guidelines, communication/marketing	National
Ayala [92]	2013	Null				R	R	QE	2 months	D, H	Guidelines, service provision, communication/marketing	Local
Geliebter [93]	2013	Positive		R				RCT+	8 weeks	$, D, H	Fiscal measures	State
Gittelsohn [94]	2013	Mixed^^			R	R		RCT+	14 months	Q, H	Service provision, communication/marketing	State
Sadler [95]	2013	Null	R					N	1 year	$	Environmental/social planning	Multilevel
Waterlander [41]	2013	Mixed^^		R		R		RCT+	6 months	$	Fiscal measures, communication/marketing	National
Bangia [96] *Bangia 2017	2014	Negative				R		QE	4 months (???)	$	Communication/marketing	Local
Cawley [97]	2014	Mixed^^				R		QE	2 years	$	Communication/marketing	National
Cummins [98]	2014	Null	R					QE	6-9 months	D, H	Guidelines, service provision, communication/marketing, environmental/social planning	Multilevel
Foster [99]	2014	Positive			R	R	R	RCT+	6 months	$	Guidelines, communication/marketing	Multilevel
Gill [47]	2014	Positive	R					N	1 year	D	Environmental/social planning	Local
Paek [100]	2014	Positive			R	R		QE	6 months	D	Guidelines, service provision, communication/marketing, environmental/social planning	Local
Papies [39]	2014	Positive				R		QE	Mean = 15 min	$	Guidelines, communication/marketing	Local
Dubowitz [101]	2015	Mixed^^^	R					RCT+	1 year	$, D, Q, H	Environmental/social planning	Multilevel
Elbel [102] *Elbel 2017	2015	Null	R					RCT+	1 year	$, D, H	Environmental/social planning	Local
Fuller [54]	2015	Positive	R					QE	1 year	$	Environmental/social planning	Local
Nikolova [103]	2015	Positive				R		N	6 months	$	Guidelines	State
Payne [104]	2015	Positive				R		RCT+	28 days	$	Communication/marketing	State
Salmon [42]	2015	Positive				R		QE	4 days	$	Communication/marketing	Local
Taillie [105]	2015	Null			W	R		N	2 years	$	Guidelines, regulation	National
Adam [57]	2016	Null					R	QE	5 weeks	$	Guidelines, fiscal measures, environmental/social planning	Multilevel
Ortega [106]	2016	Null			R	R	R	RCT+	2 years	$, E	Guidelines, communication/marketing	Local
Payne [107]	2016	Positive				R		RCT+	2 weeks	$	Communication/marketing	State
Schultz [108]	2016	Positive				R		QE	4 months	D	Communication/marketing, environmental/social planning	Local
Surkan [109]	2016	Positive		R	R	R		QE	3 months	$	Guidelines, fiscal measures, communication/marketing	Local
de Wijk [38]	2016	Null					R	QE	8 weeks	$	Environmental/social planning	Local
Winkler [56]	2016	Mixed^^				R	R	N	4 weeks	$	Communication/marketing, environmental/social planning	Local
Adjoian [110]	2017	Mixed^			R	R	R	QE	2 weeks	$	Guidelines	National
Albert [111]	2017	Null	R		R	R	R	QE	3.5 years	$, D	Legislation, regulation, environmental/social planning	Multilevel
Bangia [112] *Bangia 2014	2017	Positive				R		QE	22 min	$	Communication/marketing	Local
Brimblecombe [49]	2017	Positive		R		R		RCT+	6 months	$	Fiscal measures	National
Budd [113•]	2017	Mixed^^		R,W	R	R,W		RCT+	6 months	$	Guidelines, regulation, fiscal measures	Multilevel
Elbel [114] *Elbel 2015	2017	Null	R					QE	17 months	$, D	Fiscal measures, environmental/social planning	Local
Ferguson [51]	2017	Null		R,W		R		N	1 year	$	Fiscal measures, communication/marketing	National
Gittelsohn [115]	2017	Mixed^^			W	R		RCT+	2 years	$, Q	Service provision, communication/marketing	Local
Hobin [53]	2017	Positive				R,W		N	6 months	$	Guidelines	National
Liu [116]	2017	Positive		R		R	R	QE	4 months	D	Guidelines, fiscal measures, communication/marketing, environmental/social planning	Multilevel
Minaker [55]	2017	Positive		R	W	R	R	QE	8 months	$	Environmental/social planning	Local
Rushakoff [117]	2017	Positive			R	R		QE	18 months	$, D	Communication/marketing, environmental/social planning	Local
Toft [58]	2017	Mixed^^		R	R		R	QE	3 months	$	Fiscal measures, environmental/social planning	National
Vandenbroele [61]	2017	Positive			R			QE	1 month	$	Guidelines	National
Blake [48]	2018	Positive		R		R		QE	17 weeks	$	Legislation, service provision, fiscal measures	Multilevel
Franckle [118]	2018	Positive		R		R		RCT+	5 months	D	Guidelines, fiscal measures, communication/marketing, Environmental/social planning	Multilevel
Jilcott Pitts [119]	2018	Null	R					QE	1 month	D, H	Environmental/social planning	Local
Payne [120]	2018	Positive				R	R	QE	1 month	$	Communication/marketing, environmental/social planning	Local
Polascek [121]	2018	Positive		R				RCT+	4 months	$	Legislation, service provision, fiscal measures	Multilevel
Rogus [122]	2018	Mixed^	R		R			QE	17 months	D	Fiscal measures	Multilevel
Walmsley [43]	2018	Positive					R	N	2 years	$	Environmental/social planning	Local

Effect: specific combinations of mixed effects are indicated by ^ = positive + negative; ^^ = positive + null; ^^^ = positive + null + negative

*Geog*, geographic access; *Price*, price; *Prod*, product; *Prom*, promotion; *Place*, placement. The latter four represent the 4Ps of marketing

*R*, *W*: responsibility for implementing intervention changes; *R* = retailer and *W* = wholesaler/distributor

Study design: QE = quasi-experimental; RCT+ = randomized controlled trial or other design with comparison groups; *N* = natural experiment

Outcome(s): $ = sales or purchasing; D = dietary intakes; Q = diet quality; H = other health measures

*BCW* behavior change wheel

*Articles reporting on same intervention are listed with asterisks for cross-referencing

### Effectiveness of the Interventions in Influencing Diet-Related Outcomes

Table 2 provides an overview of the 86 included articles, including the focus of the intervention (geographic access and 4Ps), direction of effect on diet-related outcomes, and associated policy levers according to the Behavior Change Wheel. The table is organized by date of article publication in order to highlight the evolution in the literature over time.

The earliest included paper was published in 1974 [64], and the field has expanded rapidly in the last decade: 61 articles were published from 2008 to 2018. We noted a few thematic and temporal trends. The bulk of studies used promotion-based intervention strategies (sole marketing “P”). Promotion interventions have declined in relative prominence over time, with a greater proportion of studies from 2008 onward based on two or more Ps, as part of a multipronged retail intervention strategy. The literature dealing with community food environments (e.g., store openings to improve geographic access in an underserved community) remains relatively distinct from 4P interventions, with only a handful of studies combining both spatial and in-store strategies. Recent literature has incorporated food system elements, with wholesalers/suppliers as part of interventions, including responsibility for implementing changes.

Consumer purchasing, using an objective measure such as sales data, was a dietary outcome assessed in a majority of studies (52 articles, 60%) (not shown in table). Only 24 articles (28%) assessed dietary intake. Of these, five articles used a 24-h diet recall [85, 93, 101, 102, 114]; two used a 7-day food record [46, 47]; two used full food frequency questionnaires [94, 98]; and the remainder used a brief diet screener, or other brief module as part of a consumer survey.

The majority of articles (58 articles, 67%) described at least one positive effect on diet. Very few articles reported a negative effect (6 articles, of which 4 also reported a positive effect on another dietary outcome). Almost half of articles (43%) described at least one null effect.

Fifteen articles reported mixed effects: 4 articles, positive + negative; 10 articles, positive + null; and 1 article, positive, null, and negative). Note that in all instances of mixed effects, at least one positive effect of the intervention was reported. The mixed effects demonstrate the complexity of purchasing decisions, such as combined “healthy” and “unhealthy” purchases, substitution effects, and the inability to distinguish residual variation in purchasing from environmental versus individual factors. For example, Adjoian et al. [110] assessed purchasing in response to a “healthy checkout” intervention, part of a municipal government supermarket program. They found that a greater proportion of customers bought healthy snacks when using the healthy checkout versus the standard checkout. They also found that a lesser proportion of customers purchased unhealthy snacks from the healthy checkout versus the standard. It is easy to assume from these results that the environmental intervention largely “worked” and that residual unhealthy purchasing would be due to individual factors in a given checkout line. However, the team also found that over a third of items paid for at the healthy checkout were unhealthy items selected from the standard checkout.

Eleven of the 14 articles (79%) describing geographic access interventions reported a mixed or null effect. Not including the two articles using marketing strategies plus geographic access components, when considering 4P (product, promotion, placement, and price) interventions only, a comparable proportion of multi-component interventions tended to have mixed and null effects (15 of 32 articles, or 47% mixed/null), as compared to single component interventions (17 of 40 articles mixed/null, 43%, and 2/40 negative, 5%) (see Table 2).

### Policy Levers Underpinning the Interventions

The policy assumptions underpinning interventions have evolved over time. Our policy analysis was intended to unearth what policies the authors expected governments to adopt on the basis of a “successful” intervention, or to detect where authors were attentive to the existing policy context governing stores, and tailored their intervention or evaluation accordingly. As displayed in Table 2, the earlier intervention literature generally emphasized more individualized behavioral assumptions about how policy should support nutrition promotion (e.g., communications/social marketing policies). In contrast, the relative proportion of interventions with a focus on environmental and social planning modifications, and fiscal policies, has increased in the last decade.

Coding based on the NOURISHING framework (not shown in table) also showed the relative emphasis on information-based (rational actor assumptions), in contrast to environmental- and incentive-based policy levers (boundedly rational assumptions). Policy domain “S” (set incentives) in the NOURISHING framework, for instance, is explicitly about incentives and rules to support healthier retail and foodservices environments; 32 (37%) of the articles provided evidence that could be used to inform this type of policy. In contrast, the second “I” (inform people) (35%) and “G” (give nutrition education) (21%) were together even more prominent. For example, one intervention implemented an information kiosk based on a US dietary guideline campaign in-store for 12 weeks [90]. Evidence from this study could reasonably inform public policy tools for nutrition education and dietary guideline implementation, but would be unlikely to inform any government guidance for healthier retailer practices, despite being situated in the setting of the store. Economic policy instruments “U” (use economic tools) featured in just 14 articles. Only 11 articles discussed improving the quality of the food supply (first “I” of the framework), such as the need to address stores’ distributor base [86].

Thirty-four articles (40%) focused on enabling policy conditions that could be adopted through local levers; these were often interventions to address geographic access, such as economic development/urban planning. Twenty-five articles (29%) focused on national level changes, typically large-scale labeling or information initiatives by supermarkets.

The majority of papers (54 articles, 63%) did not mention any specific enabling policies or policy recommendations. Those that did (32 articles, 37%) described options such as

- Targeting business interests with locally appropriate pricing structures, marketing, branding, and stocking policy [98];
- Municipal economic development initiatives [46, 114]; including microfinancing [89] and attending to local socio-cultural context in retailing [92];
- Taxes and subsidies [48, 49, 58, 100]; and
- Shelf labeling requirements or incentives [29, 53, 109, 123].

## Discussion

The retail food environment intervention literature continues to grow and has become more robust overall, with clearer evidence of the effect of interventions on diet-related outcomes, including consumer purchasing, dietary intakes, and health. There is still much scope for development in the field to improve our understanding of the complex relationship between components of interventions and specific dietary behavior.

Retail intervention strategies have received occasional blanket criticism for a lack of effectiveness [106]. Caution has been directed especially to addressing spatial gaps in store access [98, 114, 124], and in our review, a high proportion of these interventions had mixed or null effects on diet. The combination of geographic access and in-store strategies has had minimal uptake, and we would echo others in recommending that is an area ripe for elaboration [45••, 98, 102], given the ample evidence that despite the potentially positive effects of introducing new stores in terms of food access, that merchandising activities within them can continue to represent an unhealthy influence on dietary behavior.

Confirming earlier systematic reviews, the majority of interventions showed at least one positive effect on a diet-related outcome, particularly among 4P strategies. Among 4P studies, a comparable proportion of articles reporting on multi-component strategies and single component strategies had mixed and null effects. That said, many of the multi-component studies are increasingly attempting to both intervene in and evaluate more than one dietary outcome. This is a development that has strengthened the literature but may also explain mixed outcomes. Continued engagement with multipronged interventions may offer further insights into implementation, and to improving measurement of linking steps in the impact on diet-related behavior. For example, Gittelsohn et al. evaluated the effect of a combined product availability and promotion initiative working in partnership on the Navajo Nation with stores in those communities, to assess the impact on psychosocial predictors of food selection and self-reported food purchasing practices in addition to weight [94]. This randomized controlled trial had a null result in bivariate analyses comparing intervention and comparison groups at follow-up, but found a positive effect on study outcomes mediated by exposure to the intervention.

The widespread use of proximal outcome metrics measured from sales data has benefitted the quality of the literature. The use of purchasing data linked to individuals/households (e.g., loyalty cards) is a particularly strong option. Sales/purchasing data offers researchers confidence to communicate objective study outcomes to policymakers as well as to retail business stakeholders who are interested in the direct and indirect impact of interventions on store revenue and economic viability. A continued research gap, however, is the robustness with which dietary intakes are examined. Kirkpatrick et al. [125], in a systematic review of dietary assessment in food environments research (articles from 2007 to 2012, predominantly cross-sectional assessments), cautioned that the predominance of brief dietary assessment instruments was a limitation, and contributor to measurement bias. Our review suggests that this issue persists and may have led to null or mixed effects in some cases, as well as a lack of generalizability and reproducibility. Indeed, the heterogeneity in dietary assessment, as well as other outcome measures in this review, contributed to our inability to meaningfully compare magnitude of effect among the diverse studies included. Although brief diet assessment tools are less resource-intensive—and may be adequate for focused assessment of one component of diet, the case for some interventions—on the whole, where the intent is to capture intervention effects on total diet in the short term, a 24-h recall would be less prone to systematic error [125]. Another benefit of the 24-h recall specifically in retail interventions would be to capture contextual attributes around food selection, to further unpack how the intervention response occurs in the community. For example, the 24-h recall may better reflect dietary patterns in relation to food supply given that it is not restricted to a pre-specified food lists, offering the potential to assess dietary substitutions in response to an intervention, and details of location of eating or purchase can be readily collected alongside intakes.

### Behavior Change Communication

The results of this review indicate that behavior change communication approaches are still a mainstay of retail food environment interventions for changing diets. This may be a reflection of the overall development of the field, which is still relatively new, and has drawn from disciplinary insights in marketing and consumer cognition. Another consideration is that behavior change communication strategies may be more acceptable and feasible for retailer partners to implement. Environmental and social restructuring, and fiscal interventions have become more important within the rapid expansion of retail food environment research in the last decade, likely due to the influence of behavioral economics, social epidemiology, spatial and economic geography.

From a policy standpoint, we would also argue the possibility that influencing food choices in store environments continues to be viewed as a form of “downstream” behavioral health promotion and that “upstream” public policies work separately. It is possible that researchers assessing diet-related interventions in-store see these strategies as unique from—if complementary to—the enabling public policy levers that create broader transformative change in consumption patterns at the population level. This would explain, in part, the distinctiveness of many fiscal interventions that could not be captured within our review inclusion criteria. For example, promotion strategies directed at a few products may not need to wait for a specific policy decision to implement the change widely, as long as buy-in is secured from retailer partners. In contrast, substantive adoption of successful pricing strategies may rely on longer term structural changes and public budget commitments, including adoption of development subsidies, coordination of the supplier base, tax expenditures, or other shifts in tax structures, to motivate corporations to act. This has health equity implications, where the low-hanging fruit of promotion strategies may be used more widely through targeted efforts at dietary improvement, resulting in uneven implementation at the population level.

### Policies Enabling Interventions

The studies included in our review indicate that there has been limited attention to enabling policies, or the system-wide policy context where interventions are designed and delivered. In part, this may have been an artefact of publication conventions, where contemplating policy implications that seem to stray from specific outcomes is discouraged. Studies that commented on enabling factors acknowledged that aligning with local context was important [92], which includes attending to how policies structure the behavior of actors well beyond eaters, and beyond the health sector [25•, 126, 127]. Enabling policies could encompass structures affecting store capacity and viability—such as trade pacts, labor market policies, social protections, and other forces that underpin household purchasing power. These, too, are part of the agenda-setting calculus and policy trade-offs to address diet-related risk [114]. There is a limited basis for assessing how these types of policies can link up with store-level interventions within the papers in this review. An enabling policy environment may be an important contextual feature to isolate for measurement or otherwise take into account, to assess its interaction with intervention design, implementation and outcomes [128, 129]. Adding context may have advantages for understanding why interventions succeed or fail, and how they can be adapted or translated across settings.

Most of the interventions included in this review were led by public health (including researchers and/or municipal agencies) and targeted at consumers. Very few studies targeted retailers themselves as a policy actor whose behavior could be changed in a healthier direction. The distinctive perspective of the retailer [17] has become increasingly important in measuring and attempting to modify retail food environment features to become more health-promoting. Only recently has observational research on food stores begun to focus on retailers’ role and indeed agency in shaping food access within the food system [130–133], and few interventions have been retailer-led [48, 134, 135]. Milio has argued that to make the “healthy choice the easy choice” there is a two-part health promotion imperative to direct behavior change incentives towards consumers as well as corporations [136, 137]. Although interventions are being delivered in a growing variety of community settings, the bulk of the retail evidence still comes from a body of work that largely assumes a theory of change relying on the consumer as its focus.

There are notable exceptions: Budd et al.’s article on the B’More Healthy: Retail Rewards intervention is part of a long trajectory of research programs examining multipronged small store interventions in that US jurisdiction [113•]. They focused on retailer outcomes, and considered a full spectrum of behavior change mechanisms for both retailers and consumers in producing the dietary outcome.

In the last decade, the literature has increasingly emphasized evaluating interventions within the ecosystem of the retail food environment [11, 45••], which aligns with a healthy public policy approach. Our review indicated that area-based versus in-store interventions seem to focus on a fundamentally different vision of the role of policy in healthier community environments. And this may be related to the clearer imperative for area-based interventions to situate stores relationally in the retail ecosystem. Although supermarket interventions have been carried out in various forms of cooperation with retailers over the course of the intervention literature [53, 67], we may be continuing to miss an important policy opportunity to target those retailers in more holistic health promotion efforts. The growing literature targeting wholesalers and supply chain pressures for small stores in interventions is an integrative development that may help expand our understanding of enabling policies for the retail ecosystem [51, 53, 55, 85–87, 105, 113•, 115].

### Limitations

Our review had a number of limitations. Our aim was to capture a full scope of retail food environment interventions, so unlike some past reviews, we combined geographic access and in-store approaches. Although this met our objectives, the resulting methodological complexity meant that we were unable to capture fully how multipronged interventions may have had explanations for mixed or null effects in comparison to narrower interventions that did not account for store ecosystems. This complexity and heterogeneity also limited our ability to assess and meaningfully compare effect sizes among interventions. As such, we chose to focus on reporting direction of effect, and how this differed among study types. Further analyses might consider developing meta-analysis methods isolating a subgroup of interventions with greater comparability of measures, such as the store sales data studies. Another limitation of this review was our criterion that included interventions should target a general population of shoppers, which resulted in the exclusion of a number of price-based intervention studies that have been based on membership in a cohort [31, 32]. This means that the full scope of fiscal policy discussion in the literature is missing. We excluded studies that had no dietary outcome; paradoxically, this meant that we could not capture some retailer studies that may have offered relevant insights into policy levers. Dunaway et al.’s study [138], for example, was excluded but is one of the few studies beginning to measure retailer characteristics in detail, conducting an in-depth financial analysis alongside evaluating the effect of an infrastructure project on stocking of fruits and vegetables.

## Conclusion

Retail food environments are one of the main sources of diet-related risk, but also hold health promotion policy possibility [126]. Retail stores are private corporations, but can also be considered a health promotion setting: [139, 140] a place-based organizational interface between the complex food system and eaters [141]. This review provides an update on the growing array of health promotion interventions taking place in the store environment, and their effectiveness in influencing diet-related outcomes. Retail stores are physical, social, economic, and cultural spaces that shape our dietary behaviors and where structural barriers to nutritional health such as the power over and ownership of food sources are manifest. Our review attempts to expand how we think about public policies that can support and enable effective interventions in these spaces.
